# In Vivo Optical Reporter-Gene-Based Imaging of Macrophage Infiltration of DNCB-Induced Atopic Dermatitis

**DOI:** 10.3390/ijms21176205

**Published:** 2020-08-27

**Authors:** Sang Bong Lee, Hyeonsoo Park, Jae-Eon Lee, Kil-Soo Kim, Yong Hyun Jeon

**Affiliations:** 1 Korea Institute of Medical Microrobotics (KIMIRo), Gwangju 61011, Korea; sangbongyi1@kimiro.re.kr; 2 Laboratory Animal Center, Daegu-Gyeongbuk Medical Innovation Foundation, Daegu 700-721, Korea; heonsoopark83@gmail.com (H.P.); koof12@dgmif.re.kr (J.-E.L.); kslac@dgmif.re.kr (K.-S.K.); 3 Research Center of Stickus Corporation, Haeundae-gu jaesong-dong 1050-21, Busan 48054, Korea; 4 Department of Biomaterials Science, College of Natural Resources and Life Science/Life and Industry Convergence Research Institute, Pusan National University, Pusan 50463, Korea; 5 College of Veterinary Medicine, Kyungpook National University, Daegu 700-721, Korea; 6 Leading-Edge Research Center for Drug Discovery and Development for Diabetes and Metabolic Disease, Kyungpook National University Hospital, Daegu 700-721, Korea

**Keywords:** atopic dermatitis, reporter macrophages, enhanced firefly luciferase gene (effluc), cell tracking, bioluminescence imaging (BLI)

## Abstract

This study was conducted to monitor the macrophage infiltration of atopic dermatitis (AD)-like skin lesions and to evaluate the effects of anti-AD therapeutic agents in immunocompetent mice via optical reporter-gene-based molecular imaging. The enhanced firefly luciferase (effluc)-expressing macrophage cell line (Raw264.7/effluc) was intravenously introduced into mice with 2,4-dinitrochlorobenzene (DNCB)-induced AD, followed by bioluminescent imaging (BLI). After in vivo imaging, AD-like skin lesions were excised, and ex vivo imaging and Western blotting were conducted to determine the presence of infused macrophages. Finally, the therapeutic effect of dexamethasone (DEX), an AD-modulating agent, was evaluated via macrophage tracking. In vivo imaging with BLI revealed the migration of the reporter macrophages to DNCB-induced AD-like skin lesions on day 1 post-transfer. The greatest recruitment was observed on day 3, and a decline in BLI signal was observed on day 14. Notably, in vivo BLI clearly showed the inhibition of the reporter macrophage infiltration of DNCB-induced AD-like skin lesions by DEX, which was consistent with the reduced AD symptoms observed in DEX-treated mice. We successfully visualized the macrophage migration to DNCB-induced AD-like skin lesions, proving the feasibility of macrophage imaging for evaluating AD-regulating drugs in living organisms.

## 1. Introduction

Atopic dermatitis (AD) is a global health problem, and ~20% of patients with AD throughout the world are teenagers and children [1,2]. Early onset AD occurs during the period from birth to 2 years of age, with approximately 60% of the cases starting by age 1, and 16% of the cases resolving by 12 years of age. Although AD is mainly identified as a disease of children, an increasing number of reports indicate that it is more common in adults than was previously thought, the prevalence being 3.2% and 10.2% in the adult population in the United States. Females exhibit a higher prevalence of AD than males; however, the exact mechanism related to the difference between males and females is unclear. AD caused by increased serum immunoglobulin E (IgE), inflammatory cytokine, and histamine levels is generally a chronic inflammatory skin disease, characterized by the symptoms of erythematous, pruritic, and excoriated papules [3,4,5,6,7]. For many years, therapeutic approaches for AD have been dependent on the application of local or systemic corticosteroids [8]. However, these therapeutic strategies have several side effects in AD patients. Although many attempts have been made to develop anti-AD agents for overcoming the limitations of clinical drugs currently used against AD, clinical therapeutic outcomes have remained unsatisfactory [9].

Sensitive, non-invasive, and quantitative detection of AD-associated inflammation is important for patient management and successful treatment following early diagnosis, but many difficulties remain owing to the lack of a sufficient understanding of various mechanisms underlying AD-related inflammation. Therefore, a robust and quantitative approach is urgently required for the evaluation of AD-related inflammation in living organisms.

Several studies have shown that macrophages are strongly associated with the pathogenesis of AD [10,11,12]. After the induction of AD, macrophages accumulate acutely and chronically in inflamed lesions. Furthermore, they interact closely with various inflammatory immune cells, such as mast and T cells, thereby leading to the aggressive progression of severe AD. However, the exact mechanism of macrophage activation has not been fully explored. Thus, there is a need to comprehensively explore the biological behavior of macrophages for a better understanding of their role in the complex AD network.

**Figure 1:**
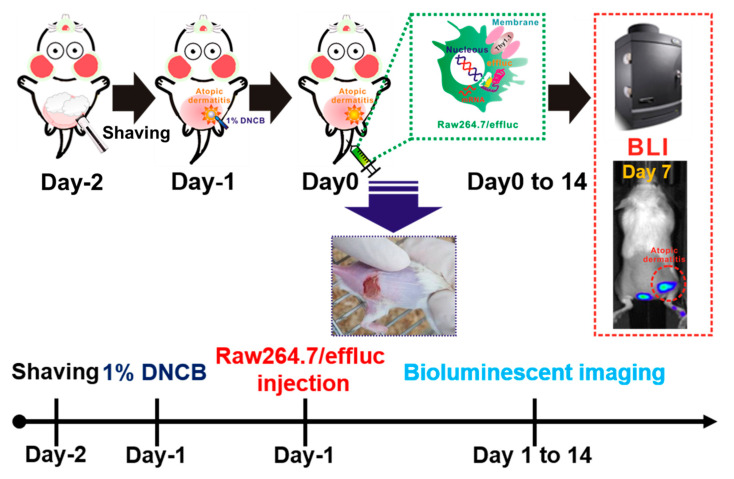
Scheme for the in vivo tracking of the macrophage infiltration in dinitrochlorobenzene (DNCB)-induced atopic dermatitis (AD) using optical reporter-gene-based molecular imaging. Mice were anesthetized with 1%–2% isoflurane gas, and then the dorsal site of the mice was carefully shaved prior to DNCB application. Reporter macrophages were injected via the tail vein, and bioluminescence imaging (BLI) was performed with the optical imaging system.

Molecular and genetic imaging modalities have been studied to visualize the localization, proliferation, and migration of immune, stem, and cancer cells [13,14,15,16]. Among various reporter-gene-based imaging approaches, in vivo bioluminescence imaging (BLI) using optical reporter genes including firefly, *Gaussia*, NanoLuc, and *Renilla* luciferases has been widely applied for in vivo cell tracking owing to its high sensitivity and ease of use [16,17,18]. Hence, to improve our understating of the complex biological effects of macrophages on AD-associated inflammation, we selected the highly sensitive enhanced firefly luciferase (effluc) optical imaging approach for in vivo macrophage tracking. This molecular imaging approach has been found to be suitable for the visualization of macrophage distribution in various acute and chronic inflammation models. For an animal disease model, dinitrochlorobenzene (DNCB)-induced AD was used in this study, because DNCB-induced AD exhibits drastic induction in IL-1β and TNF-α inflammatory cytokines at mRNA and protein levels, with T-cell activation and infiltration of neutrophil and monocytic cells. Moreover, DNCB-induced inflammation lesions are similar to the clinical symptoms of AD-like lesions, including erythema/hemorrhage, edema/excoriation, erosion, scarring/dryness, and lichenification. For the tracking of the macrophage infiltration to DNCB-induced AD lesions, immunocompetent mice with DNCB-induced AD received reporter macrophages via tail vein injection, and their migration into AD lesions was serially monitored using the optical imaging system, followed by ex vivo imaging of the excised AD lesions. Finally, using our macrophage imaging platform for the DNCB-induced AD model, we attempted to evaluate the therapeutic effects of dexamethasone (DEX), an anti-inflammatory agent, in mice with DNCB-induced AD (Figure 1).

## 2. Results

### 2.1. Expression of the Effluc Gene as an Optical Reporter Gene in Reporter Macrophage Raw264.7/Effluc Cells

**Figure 2:**
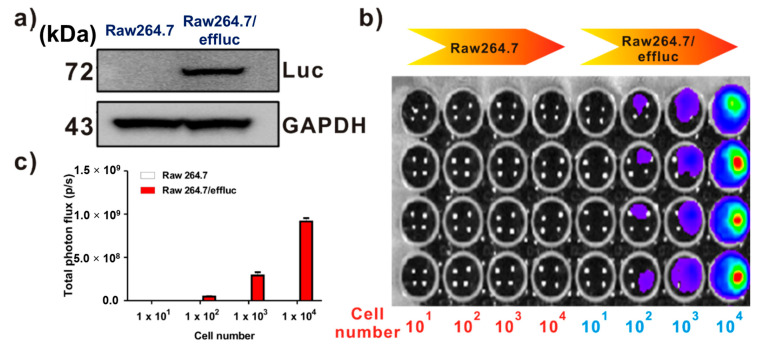
Expression of the enhanced firefly luciferase (effluc) gene as an optical reporter gene in Raw264.7 cells. (**a**) Western blot analysis showing the luciferase protein expression with anti-luciferase antibodies. (**b**) In vitro BLI in Raw264.7 and Raw264.7/effluc cells. (**c**) Quantification of the bioluminescence imaging signals. The experiments were performed at least in triplicate, and the bar graphs represent means ± SDs.

Effluc-gene-expressing reporter macrophages have been previously demonstrated [13]. Briefly, because the effluc gene is co-expressed with the Thy1.1 gene as a surrogate marker, we can easily analyze its expression levels using fluorescence-activated cell sorting (FACS) analysis with the anti-Thy1.1 antibody. The results of FACS demonstrated higher levels of effluc gene reporter macrophages. Immunoblotting with the anti-luciferase antibody showed the 72 kDa luciferase protein in Raw264.7/effluc cells, but not in parental cells (Figure 2a). In vitro BLI revealed an increase in bioluminescence signals in a cell-number-dependent manner in Raw264.7/effluc cells, but not in parental cells (Figure 2b,c).

### 2.2. Tracking the Raw264.7/Effluc Cell Infiltration of AD-Like Lesions

**Figure 3:**
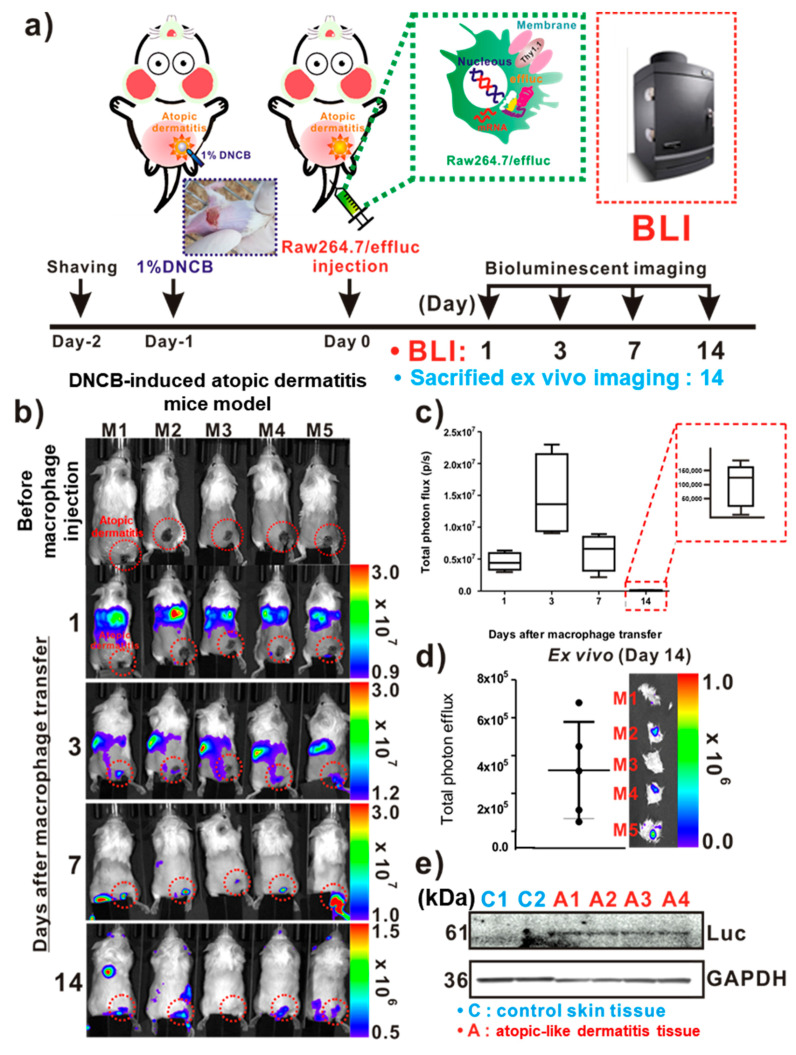
In vivo optical imaging of the macrophage infiltration of dinitrochlorobenzene (DNCB)-induced atopic dermatitis (AD). (**a**) Experimental procedures for in vivo animal experiments. (**b**) Visualization of the macrophage cell migration to DNCB-induced AD-like lesions. AD mice received reporter macrophage cells via intravenous injection, and their migration to the AD-like lesions was monitored at the indicated times. (**c**) Quantification of the bioluminescence imaging (BLI) signals in AD lesions at indicated times. Inset with square dotted lines shows the BLI signals in AD-like lesions on day 14. (**d**) Ex vivo BLI for the detection of infiltrated reporter macrophages in AD on day 14. (**e**) Immunoblot analysis in excised AD-like and control skins with anti-luciferase antibodies for evaluation of the presence of infiltrated reporter macrophages in AD-like lesions. Each group consisted of four mice, and experiments were performed at least in triplicate. The values indicate means ± SDs.

For the in vivo tracking of the reporter macrophage infiltration to AD-like lesions, a DNCB-induced atopic model was adopted in immunocompetent mice (Figure 3a). On day 1 post-DNCB treatment of the skin, AD-like lesions were clearly observed by inspection (Figure 3a inset). As shown in Figure 3b, the distribution of the migrated Raw264.7/effluc was evident in the lungs immediately after the injection of reporter macrophages, and so was their migration to the liver. The infiltration of Raw264.7/effluc into the AD-like lesions was first detected on day 3 post-atopic disease induction, and the BLI signals were detectable by day 14 (Figure 3b,c). Excised DNCB-induced skin samples also showed distinct BLI signals in AD lesions (Figure 3d, day 14 post-AD induction). Immunoblot analysis consistently showed the expression of effluc in AD-like lesions, but not in normal control skin samples (Figure 3e).

### 2.3. Feasibility of Reporter Macrophage Imaging for the Evaluation of the AD-Modulating Agent

**Figure 4:**
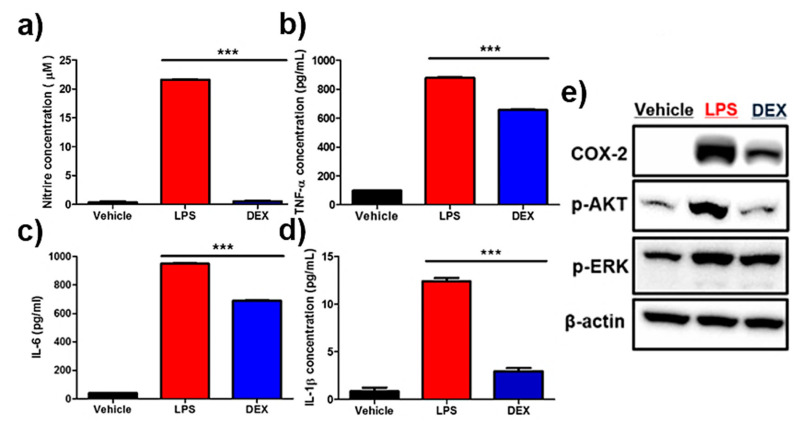
Effects of dexamethasone (DEX) on lipopolysaccharide (LPS)-stimulated reporter macrophages. Reporter macrophages were pre-treated with the indicated concentration of DEX for 1 h and then with LPS (1 μg/mL, 24 h). (**a**) Nitric oxide (NO) levels in respective reporter macrophages. Expression levels of the cytokines (**b**) TNF-α, (**c**) IL-6, and (**d**) IL-1β in the culture medium were determined by the enzyme-linked immunosorbent assay (ELISA). (**e**) Western blot analysis showing the expression levels of COX-2, p-AKT, and p-ERK in respective reporter macrophages. Values obtained from three individual experiment are expressed as the mean ± standard deviation (SD), *** *p* < 0.001

Nitric oxide (NO) assay showed a marked increase in NO levels in lipopolysaccharide (LPS)-treated reporter macrophages, and their inhibition was clearly detectable in DEX-treated cells (Figure 4a). Accordingly, LPS treatment led to a drastic increase in anti-inflammatory cytokines such as IL-6, IL-1β, and TNF-α (Figure 4b–d). Conversely, the reversal of upregulated pro-inflammatory cytokines was observed in DEX-treated reporter macrophages. Next, the protein levels of pro-inflammatory response-associated factors, including COX-2, p-AKT, and p-ERK, were analyzed by Western blot analysis. As shown in Figure 4e and Figure S1, an increased protein expression of COX-2, p-AKT, and p-ERK was downregulated in reporter macrophages by DEX treatment.

**Figure 5:**
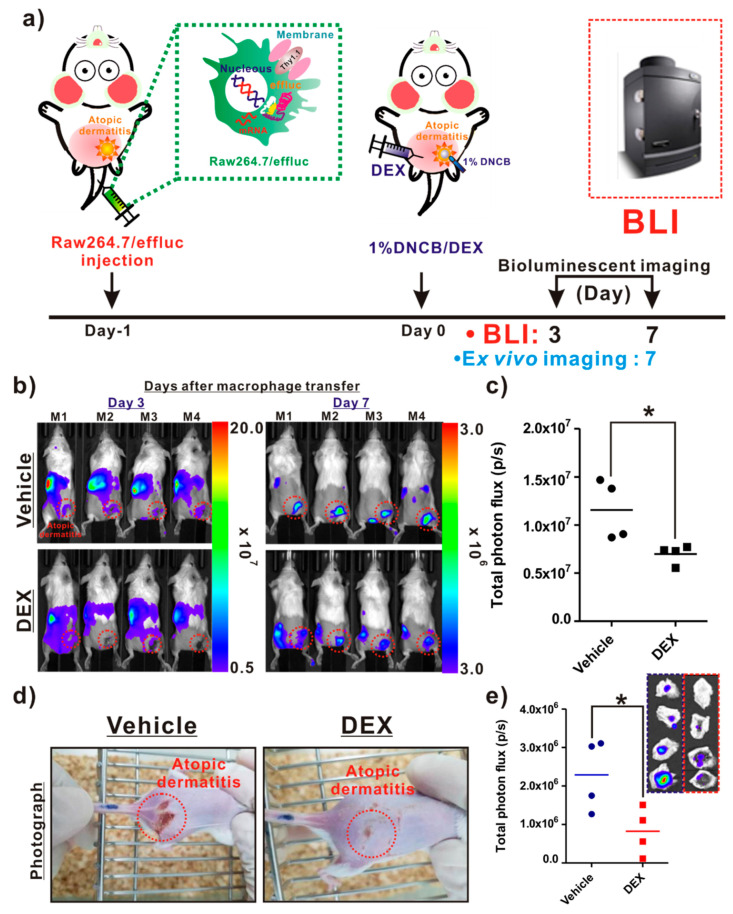
In vivo assessment of the therapeutic effects of an atopic dermatitis (AD)-modulating agent using the macrophage tracking approach. (**a**) Protocols for in vivo animal experiments. (**b**) In vivo optical imaging of the macrophage migration into AD-like lesions in the vehicle- and dexamethasone (DEX)-treated mice. The dotted red circles indicate the AD-like lesions. (**c**) Quantification of the bioluminescence imaging (BLI) signals (vehicle and DEX) in AD-like lesions. (**d**) Photograph of AD mice. (**e**) Quantification of the BLI signals in excised AD-like lesions from each group. Values obtained from three individual experiment are expressed as the mean ± standard deviation (SD), * *p* < 0.05

As illustrated in Figure 5a, the following procedures were conducted for the in vivo evaluation of the anti-inflammatory agent. The infiltration of reporter macrophages was clearly detected in the vehicle group on day 3 post-induction of AD, and the BLI signals in AD-like lesions were detected up to day 7. However, DEX treatment resulted in a crucial inhibition of the reporter macrophage movement to the AD lesions up to day 7 (Figure 5b, bottom panel). The BLI signals from AD-like lesions were significantly lower in the DEX-treated mice than in the vehicle groups (Figure 5c). Additionally, consistent with the optical imaging data, the photographs of the AD lesions showed a marked relief from AD in the DEX-treated mice, but not in the vehicle-treated mice (Figure 5d). Ex vivo imaging consistently showed a reduction in BLI signals from AD-like lesions in the DEX-treated mice compared with the vehicle-treated mice (Figure 5e).

## 3. Discussion

Macrophages are crucial immune cells associated with various pathologic processes, as well as with the development of various inflammatory diseases [19]. In particular, because macrophages include various factors that control the release of cytokines and chemokines in AD, they play an essential role in acute and chronic inflammation related to AD [20,21,22,23]. Recent studies have shown that the infiltration of macrophages into AD lesions can be a desirable therapeutic method for controlling AD-related inflammation [24] in preclinical models.

Optical reporter-gene-based bioluminescence imaging has generally been investigated for monitoring the biological behavior of various cells of interest, such as normal, cancer, and immune effector cells, owing to its high sensitivity and lack of requirement for complex synthesis procedures [16,17,18]. Among various optical reporter genes, the enhanced firefly luciferase (effluc) gene has been extensively investigated for tracking immune cells in living mice with various diseases because it can be used to detect a small number of injected immune cells in vivo. Based on these reasons, we adopted this reporter for the non-invasive and repetitive tracking of the macrophage infiltration of AD-like lesions in mice. DNCB, which has been applied in several AD-related drug development studies, was selected to induce stable clinical AD-like skin diseases in the mice, and to uncover the resulting macrophage infiltration mechanism [25,26,27]. Similar to other reports, when DNCB was applied to the skin, aggressive AD symptoms such as severe erythema, erosion, and dryness were induced. To determine the macrophage infiltration of AD-like lesions, reporter macrophages were intravenously injected into mice with AD. We observed the early distribution of reporter macrophages in the lung, and their serial migration to the liver and spleen. Interestingly, owing to the highly sensitive optical reporter genes, we also observed the first reporter macrophage infiltration of AD-like lesions on day 3 post-transfer and monitored their localization in AD-like lesions on day 14, which was consistent with the results of ex vivo imaging of DNCB-treated lesions and immunoblotting analysis using anti-luciferase antibodies. These results clearly demonstrate that optical reporter gene imaging of the effluc gene is a feasible approach for real-time monitoring of macrophage infiltration in mice with AD.

Reporter-gene-based macrophage imaging should be applied for the repetitive and quantitative evaluation of the therapeutic outcomes of disease-modulating agents in living animals. The introduction of exogenous reporter genes can affect the biological function of immune cells of interest; hence, their unique functions should be carefully examined prior to in vivo immune cell imaging of various areas. We also investigated whether reporter macrophages showed the production of inflammation mediators, pro-inflammatory cytokines, and changes in inflammatory signals in response to LPS stimulation with or without anti-inflammatory agents such as DEX. Furthermore, inflammatory responses induced by various microbial and viral infections led to the production of pro-inflammatory cytokines such as TNF-α, IL-6, and IL-1β in macrophages via upregulation of the PI3K/AKT and MAPK pathways [28]. When reporter macrophages were stimulated with LPS, we observed increased NO, as well as pro-inflammatory cytokines, in the reporter macrophages, which were inhibited by DEX. Furthermore, upregulation of inflammatory signaling pathways, including COX-2, p-AKT, and p-ERK, was clearly observed in reporter macrophages, but this was reversed by DEX. Finally, we attempted to investigate the feasibility of our macrophage tracking approach for evaluating AD-regulating agents in vivo. Most importantly, we clearly observed the inhibition of the reporter macrophage infiltration of AD lesions in DEX-treated mice, which corresponded with ex vivo BLI outcomes; consistent with in vivo BLI results, AD symptoms were relieved in DEX-treated mice. These findings suggest that macrophage tracking is a useful tool for the evaluation of AD treatment agents in living subjects.

## 4. Materials and Methods

### 4.1. Nitric Oxide (NO) Levels and Cytokine Assay

Raw264.7/effluc macrophages (1 × 10^5^ cells/well) were seeded in a 24-well culture plate and cultured for 12 h. The cells were pre-treated with 10 μM DEX for 1 h and then co-incubated with 1 μg/mL lipopolysaccharides (LPS) for 24 h. NO concentrations in the culture medium were determined by the Griess assay (Promega, Medison, Wiscosin, USA). Griess reagent (100 μL) was added to medium supernatants (50 μL) and then incubated at 37 °C for 15 min in the dark. Absorbance was measured at 520 nm. NO concentrations were calculated using 0 to 100 μM sodium nitrate standards. TNF-α, IL-6, and IL-1β expression levels in the culture medium were quantified using ELISA kits (R&D Systems, Minneapolis, MN, USA).

### 4.2. Western Blot Analysis 

Raw264.7/effluc cells were treated with or without DEX for 1 h and then with 1 μg/mL LPS for 24 h. They were washed twice with cold PBS and lysed with RIPA buffer containing a complete protease inhibitor cocktail and phosphate inhibitor (Roche, Basel, Switzerland). Equal amounts of proteins were loaded in each lane and resolved by 4%–12% gradient Bis-Tris gels (Invitrogen, Calrlsbad, CA, USA). Proteins were transferred onto 0.2 μm PVDF membranes (Invitrogen, Calrlsbad, CA, USA). The membranes were incubated overnight at 4 °C with the following primary antibodies: COX-2 (Cayman; dilution 1:3000), p-AKT (Cell Signaling, Danvers, MA, USA; dilution 1:1000), p-ERKphospho-p44/42 MAPK (phosphorylated extracellular-signal-regulated kinase (pERK1/2)) (Cell Signaling; dilution 1:2000), β-actin (Cell Signaling; dilution 1:3000). The membranes incubated with the primary antibody were then incubated with an HRP-conjugated secondary antibody at room temperature. ECL-Plus (Millipore, Burlington, MA, USA) was used to detect peroxidase activity according to the manufacturer’s protocol.

Murine macrophage Raw264.7 cells were cultured in Dulbecco’s modified Eagle medium supplemented with 10% fetal bovine serum and 1% antibiotic-antimycotic (Invitrogen, Carlsbad, CA, USA) at 37 °C in a 5% CO_2_ atmosphere. The establishment of Raw264.7/effluc cells was described previously [29].

### 4.3. Animals

Pathogen-free 6-week-old female BALB/c mice were obtained from SLC Inc. (Shizuoka, Kotoh, Japan). All the animals were cared for in accordance with the Guide for the Care and Use of Laboratory Animals of the National Institutes of Health Publication (No. 85–23, revised 2011, 8th edition). The experimental protocols were approved by the Committee for the Handling and Use of Animals at the Laboratory Animal Center, Daegu-Gyeongbuk Medical Innovation Foundation (approval number (IACUC) DGMIF-1702605-00). In vivo animal experiments were performed on female BALB/c mice weighing 25–30 g. The mice were kept at a constant temperature of 23 °C and under 55% relative humidity and a 12 h light/dark cycle. The mice were fed basic laboratory food, and water was provided adlibitum.

### 4.4. Animal Model of Atopic Dermatitis

The procedure for the development of the model was started by carefully shaving off the hair from the dorsal skin region with a fine electric shaver. Hair removal cream was applied if required to remove the remaining hair. To sensitize the skin and for the induction of atopic dermatitis, 200 μL of 1.0% DNCB in 3:1 (*v*/*v*) acetone–olive oil solution was topically applied to the exposed skin for 1 day. After the visual confirmation of the optical imaging and parameters for skin sensitization, the mice were treated with the anti-inflammation drug.

### 4.5. Animal Experiments

#### 4.5.1. In Vivo Imaging Procedure

For in vivo BLI, the mice received D-luciferin via intraperitoneal injection. BLI was performed for 10 min (effluc) after injection using the IVIS Lumina III imaging system. Grayscale photographic images and bioluminescent color images were superimposed using the LIVINGIMAGE (version 2.12, Waltham, MA, USA, PerkinElmer) and IGOR Image Analysis FX software (WaveMetrics, Lake Oswego, OR, USA). BLI signals are expressed in units of photons per cm^2^ per second per steradian (p/cm^2^/s/sr). All the mice were anesthetized using 1%–2% isoflurane gas during imaging.

#### 4.5.2. Study 1

The experimental scheme for evaluating induced-AD imaging in BALB/c mice via macrophage tracking is described in Figure 3a. Reporter macrophages (5 × 10^6^) were injected in BALB/b mice (*n* = 5) with AD-like skin lesions through intravenous injection, and in vivo BLI was performed at designated times (days: 1, 3, 7, and 14) following the transfer of reporter macrophages. After BLI, the mice were sacrificed and AD-like lesions were excised, placed on a black sheet, and subjected to BLI ex vivo. After ex vivo imaging, immunoblot analysis was performed using excised AD-like and control skin samples with anti-luciferase antibodies for evaluation of the presence of infiltrated reporter macrophages in AD-like lesions.

#### 4.5.3. Study 2

The experimental scheme for evaluating anti-AD therapeutic effects via reporter gene macrophage tracking is described in Figure 5a. On day 1, after the injection of reporter gene macrophages, the BALB/c mice were divided into two groups, namely, vehicle (*n* = 4) and DEX (*n* = 4). An AD stimulus was generated using the same procedure as described above (Study 1). A single dose of 10 mg/kg DEX or vehicle was administered to the mice with AD. BLI was conducted to determine the recruitment of reporter gene macrophages in AD-like lesions at the indicated times (days 3 and 7). After BLI, the AD mice were sacrificed and AD-like lesions were excised, placed on a black sheet, and subjected to BLI ex vivo.

### 4.6. Statistical Analysis

All data are presented as means ± standard deviation from at least three representative experiments. Statistical significance was determined using the unpaired Student’s *t*-test. *p*-Values < 0.05 were considered statistically significant.

## 5. Conclusions

We report the visualization of the macrophage infiltration of DNCB-induced AD-like lesions using the highly sensitive optical reporter gene effluc. Notably, we successfully demonstrate the feasibility of our macrophage tracking technique for evaluating AD-modulating agents in AD animal models. We believe that our macrophage imaging approach would be useful for investigating AD-associated mechanisms and evaluating novel anti-AD compounds with various pharmacodynamic effects.

## Supplementary Materials

Supplementary materials can be found at https://www.mdpi.com/1422-0067/21/17/6205/s1.

## Abbreviations

AD	Atopic dermatitis
BLI	Bioluminescence imaging
IL	Interleukin
TNF	Tumor necrosis factor
