# Supplementary material for: In Vivo Optical Reporter-Gene-Based Imaging of Macrophage Infiltration of DNCB-Induced Atopic Dermatitis
Source: Int J Mol Sci. 2020 Aug 27;21(17):6205. doi: 10.3390/ijms21176205 (PMC7503337; doi:10.3390/ijms21176205)
Supplement: Supplementary file 1 [file ijms-21-06205-s001.pdf]

**Supplementary Materials**

*Article*

# In Vivo Optical Reporter-Gene-Based Imaging of Macrophage Infiltration of DNCB-Induced Atopic Dermatitis

**Sang Bong Lee [1,†], Hyeonsoo Park [2,†], Jae-Eon Lee [2,3], Kil-Soo Kim [2,4] and Yong Hyun Jeon [2,5,*]**

1 Korea Institute of Medical Microrobotics (KIMIRo), Gwangju 61011, Korea; sangbongyi1@kimiro.re.kr
2 Laboratory Animal Center, Daegu-Gyeongbuk Medical Innovation Foundation, Daegu 700-721, Korea; jeon9014@dgmif.re.kr (H.P.); koof12@dgmif.re.kr (J.-E.L.); kslac@dgmif.re.kr (K.-S.K.)
3 Department of Biomaterials Science, College of Natural Resources and Life Science/Life and Industry Convergence Research Institute, Pusan National University, Pusan zip code, Korea
4 College of Veterinary Medicine, Kyungpook National University, Daegu 700-721, Korea
5 Leading-Edge Research Center for Drug Discovery and Development for Diabetes and Metabolic Disease, Kyungpook National University Hospital, Daegu 700-721, Korea
* Correspondence: jeon9014@gmail.com; Tel.: +82-53-790-5726
† These authors contributed equally to this work.

---

**Cells.** Murine macrophage Raw264,7 cells were cultured in Dulbecco's modified Eagle medium supplemented with 10% fetal bovine serum and 1% antibiotic-antimycotic (Invitrogen, Carlsbad, CA) at 37$^{o}$C in a 5% $CO_2$ atmosphere. The establishment of Raw264.7/effluc cells has been described in previous reports [1].

**Animals.** Pathogen-free 6-week-old female BALB/c mice were obtained from SLC Inc. (Shizuoka, Japan). All animals were cared for in accordance with the Guide for Care and Use of Laboratory Animals of National Institutes of Health Publication (No.85–23, revised 2011, 8th edition). This study experimental protocols were approved by the Committee for the Handing and Use of Animal at Laboratory Animal Center, Daegu-Gyeoungbuk Medical Innovation Foundation. (Approval number (IACUC): DGMIF-1702605-00). In vivo animal experiments were performed on female Balb/c mice weighing 25~30 g. Mice were kept at constant temperature of 23 ℃ and humidity 55% with a 12h light/dark cycle. Mice were fed with basic laboratory food and water provided *adlibitum*.

**Animal model of atopic dermatitis.** In this study, the procedure was started by carefully shaving the hair off from the dorsal skin region with a fine electric shaver. Apply hair removing cream if required to remove the rest of the hair. In order to sensitize the skin and for the induction of atopic dermatitis, 200 µL of 1.0% DNCB in 3:1 (*v*/*v*) acetone / olive oil solution was topically applied the exposed skin for one day. After the visual confirmation of optical imaging and parameters for skin sensitization, mice were treated with anti-inflammation drug.

**Animal experiments**

**In vivo imaging procedure.** For In vivo BLI, mice received D-luciferin via intraperitoneal injection. BLI was performed at 10 min (effluc) after substrate injection using the IVIS Lumina III imaging system. Grayscale photographic images and bioluminescent color images were superimposed using LIVINGIMAGE (version 2.12, PerkinsElmer) and IGOR Image Analysis FX software (WaveMetrics, Lake Oswego, OR). BLI signals are expressed in units of photons per $cm^2$ per second per steradian ($P/cm^2/s/sr$). All mice were anestherized using 1%-2% isoflurane gas during imaging.

**Study 1.**

The experimental scheme for evaluating induced AD (atopic dermatitis) imaging in Balb/c mice via macrophage tracking is described in Figure 3a. $5\times10^6$ reporter macrophages in Bal/b mice (*n* = 5) with AD-like skin lesions *via* intravenous injection, and In vivo BLI was acquired at designated times (Days: 1,3,7 and 14) following the transfer of reporter macrophages. After BLI imaging, the AD mice sacrificed and AD-like lesions were excised placed a black sheet, and subjected to BLI ex vivo. After ex vivo imaging, immunoblotting analysis confirmed in excised AD-like and control skins with anti-luciferase antibodies for evaluating the presence of infiltrated reporter macrophages in AD-like lesions.

**Study 2**

The experimental scheme for evaluating anti-AD therapeutic effects via reporter gene macrophage tracking is described in Figure 5a. On day 1, after the injection of reporter gene macrophage, the Balb/c mice were divided into two groups, vehicle (*n* = 4) and DEX (*n* = 4). AD stimuli were generated using the same procedure described above (Study 1). A single dose of 10 mg/kg DEX or vehicle was administered to mice with AD. BLI was conducted to determine the recruitment of reporter gene macrophage in AD-like lesions at the indicated times (Day 3 and 7). After BLI imaging, the AD mice sacrificed and AD-like lesions were excised placed a black sheet, and subjected to vehicle and DEX groups of BLI ex vivo.

1 Bu, L. *et al.* Outcome Prediction and Evaluation by Imaging the Key Elements of Therapeutic Responses to Cancer Immunotherapies Using PET. *Current Pharmaceutical Design* (2020).

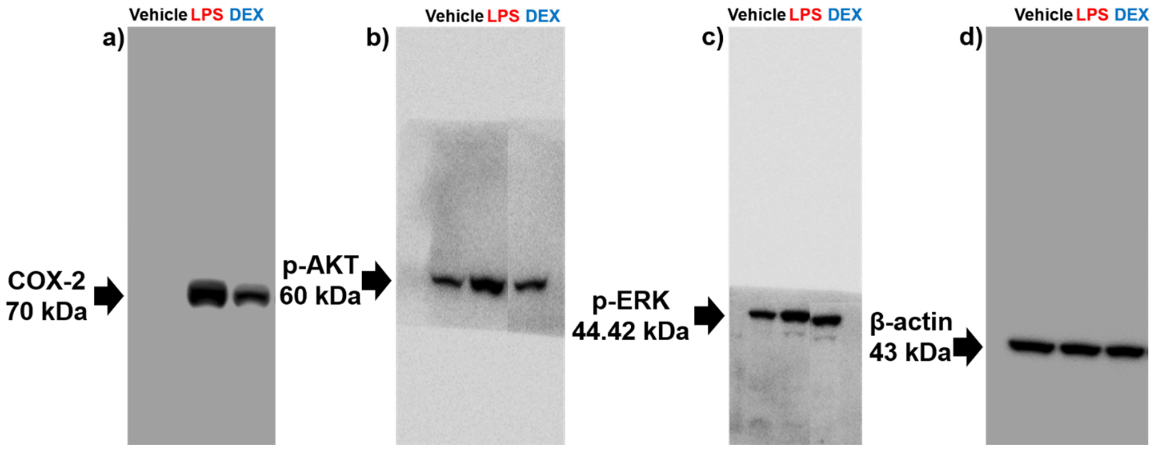


**Figure S1.** Full COX-2 (**a**), p-ARK (**b**), p-ERK (**c**), and β-actin (**d**) western blots form Fig 4 (e). Sample were run on four different membranes.
